# Modified fourth lumbar artery local perforator flap: an alternative for reconstruction of nonhealing lumbosacral spinal defects

**DOI:** 10.1186/s12893-023-01909-8

**Published:** 2023-01-13

**Authors:** Runlei Zhao, Xinling Zhang, Xin Yang, Zhenmin Zhao

**Affiliations:** Department of Plastic Surgery, Peking University Third Hospital, 49 North Garden Road, Haidian District, Beijing, 100191 People’s Republic of China

**Keywords:** Modified fourth lumbar artery local perforator flap, Superior gluteal artery perforator, Lumbosacral spinal defects, Reconstruction

## Abstract

**Background:**

The reconstruction of nonhealing lumbosacral spinal defects remains a challenge, with limited options. The aim of this article was to review the authors’ technique and experience with the modified fourth lumbar artery local perforator (MFLALP) flap for the coverage of nonhealing lumbosacral defects after spinal surgery.

**Methods:**

Between August 2012 and May 2021, we reviewed all MFLALP flaps performed for lumbosacral spinal defects. Patient demographics, wound aetiologies, surgical characteristics, and outcomes were reviewed retrospectively.

**Results:**

A total of 31 MFLALP flaps were performed on 24 patients during the research period. The median flap size was 152 cm^2^ (range, 84–441 cm^2^). All flaps survived successfully, although there were two cases of minor complications. One patient had a haematoma and required additional debridement and skin grafting at 1 week postoperatively. The other patient suffered wound dehiscence at the donor site at 2 weeks postoperatively and required reclosure. The follow-up time ranged from 6 months to 5 years.

**Conclusions:**

The MFLALP flap has the advantages of a reliable blood supply, sufficient tissue bulk and low complication rate. This technique is an alternative option for the reconstruction of nonhealing lumbosacral spinal defects.

**Supplementary Information:**

The online version contains supplementary material available at 10.1186/s12893-023-01909-8.

## Introduction

The reconstruction of posterior soft-tissue defects resulting from complications of spinal surgery is challenging for surgeons, especially when wound infection and hardware salvage are involved. This type of wound is often characterized by large subcutaneous tissue voids and dead spaces. Various vascularized flaps could be applied as a solution by enhancing vascular perfusion and reducing underlying dead space in the region. Multiple techniques for lumbosacral spinal reconstruction have been described, ranging from local perforator-based fasciocutaneous flap coverage to complex free flap reconstruction with vein grafts. Among them, paraspinous muscle flaps, gluteus maximus muscle flaps, and perforator flaps such as superior gluteal artery perforator (SGAP) and lumbar artery perforator (LAP) flaps are usually used [1–5]. However, nonhealing lumbosacral defects remain challenging to treat when the abovementioned flaps are not optimal choices for various reasons. A number of anatomical studies have suggested that the LAP and the SGAP are two major perforasomes in the lumbosacral region. These two perforasomes have massive interconnections between them, providing possibilities for new types of flaps in this region [6–9]. The purpose of this report is to review the experience with a novel flap, the local modified fourth lumbar artery perforator (MFLALP) flap, for the coverage of nonhealing lumbosacral defects after spinal surgery at our institution.

## Materials and methods

This was a retrospective study that included consecutive cases of nonhealing lumbosacral wounds from August 2012 to May 2021 at a single institution. Details pertaining to the patient age and sex, wound aetiology, presence of internal hardware, wound culture result, surgical details, complications, and follow-up time were collected from patients’ medical records. The Institutional Review Board (IRB) of Peking University Third Hospital approved the study (IRB number M2020576), and informed consent was obtained from all patients.

### Operative technique

Thorough and deliberate debridement was performed before flap dissection during each surgery. After removing unhealthy tissue, saline and 3% hydrogen peroxide were used sequentially to remove debris and anaerobic bacteria. Then, 0.5% betadine was applied and allowed to soak the wound bed and hardware for 5–10 min. Finally, a large quantity of saline, e.g., as much as 3000–6000 ml, was used for irrigation in each case. For patients with hardware, pressure irrigation was performed using a 19-gauge needle adapted to a 50-ml syringe.

**Fig. 1:**
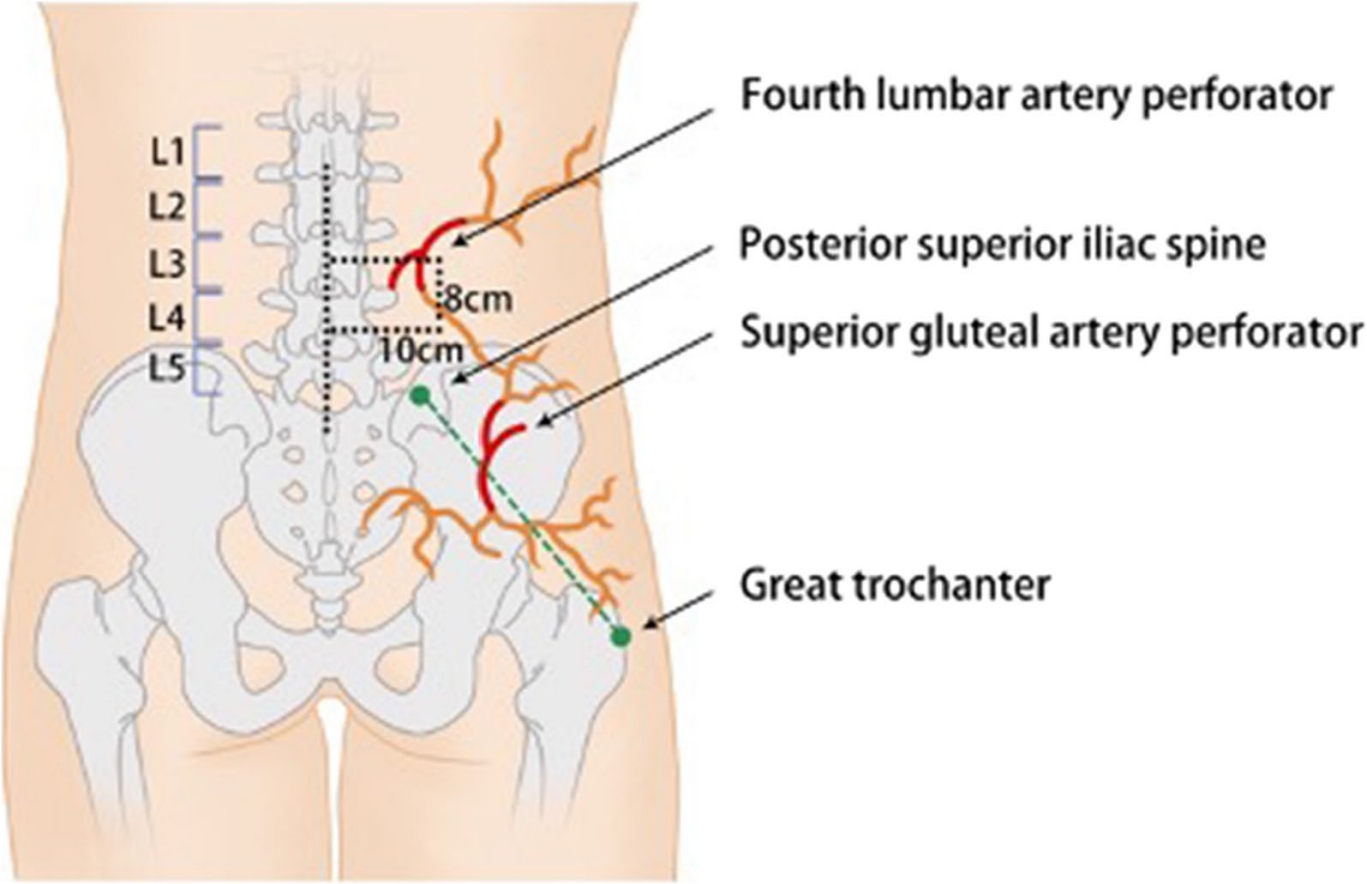
Anatomy of the fourth LAP, SGAP and their anastomosis

**Fig. 2:**
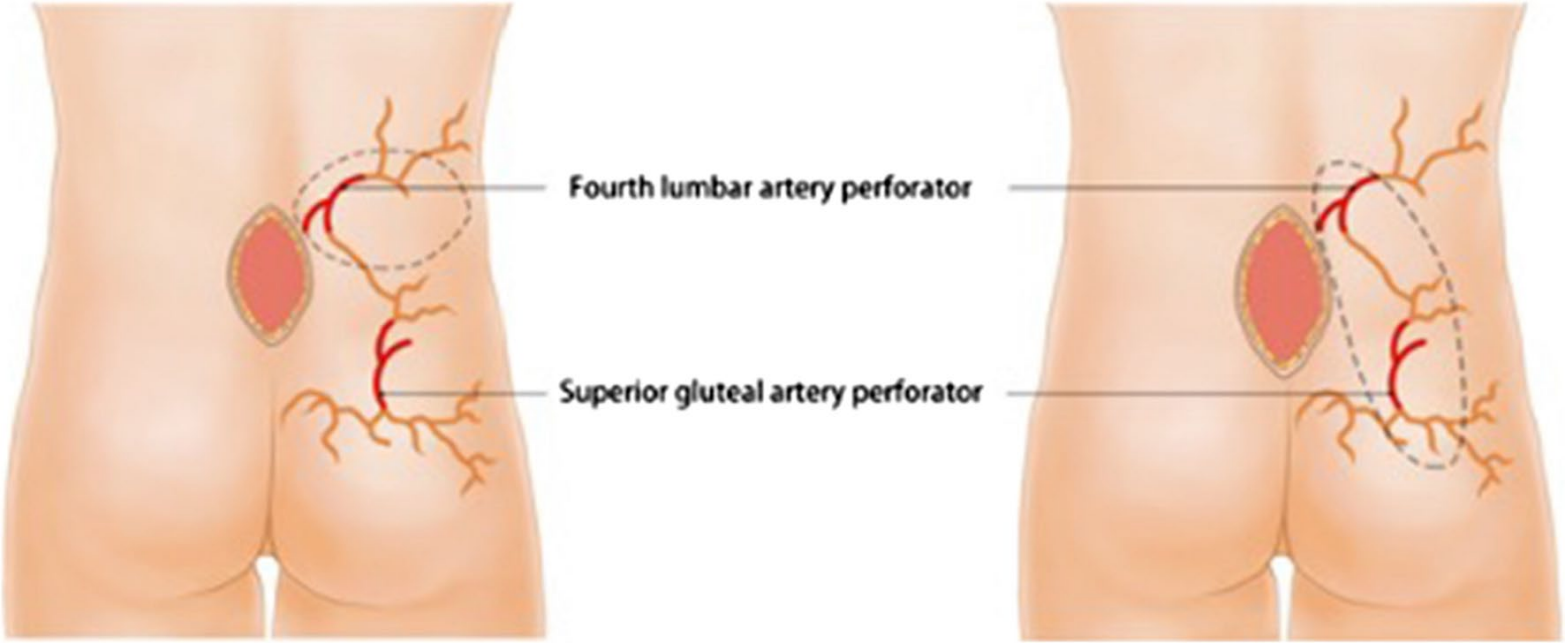
Design of the traditional fourth LAP flap (left) and the MFLALP flap (right)

The fourth LAP usually pierces the lumbar fascia between the boundary of 8 cm superior to the L4 vertebra and 10 cm lateral to the midline. The SGAP is usually located along the superior 2/3 of a line drawn from the superior posterior iliac spine to the greater trochanter (Fig. 1). A pencil Doppler flow detector was used to confirm the location of both perforators. A line was drawn between the two perforators as the axis of the flap (Fig. 2). The fourth LAP served as the pivot point, and flaps were designed based on the various wound shapes. In general, it is advisable to include the bulk soft tissue at the distal part of the flap to provide a sufficient amount of tissue volume to fill in the dead space of the wound. The flap can then be elevated from the distal part on the deep fascia layer, in which the fascia should be included to protect the perforators and the cutaneous nerve. The incision was made outward from the superficial to deep layer to include more fasciocutaneous tissue in the flap. The superior cluneal nerve usually runs along the fourth LAP and should be included in the flap to make it a sensory flap whenever possible. Small perforators were made along the pathway to harvest the flap; it was important to ensure that the pedicle of the flap was not too narrow. After confirmation of the perfusion of the flap, it was transferred to the recipient site. De-epithelization was performed to match the dead space within the wound if necessary. The donor site was typically closed; however, on occasion, a skin graft was applied when necessary. The “mismatch” technique was used when the flap was stitched to the recipient site. After the flap was oriented, each stitch was sewn 5–10 mm farther on the recipient side. We used this technique to minimize the tension of the distal part of the flap. A closed suction drain was placed at both the donor and recipient sites. Prophylactic antibiotics were given to the patient for 3–7 days postoperatively, and patients were advised to sleep on their stomach or side to avoid excessive pressure on the flap. Patients were encouraged to ambulate as soon as possible, and the sutures were removed two weeks postoperatively.

## Results

### Patient characteristics

A total of 31 MFLALP flap-related surgeries were performed on 24 patients, including 17 unilateral cases and 7 bilateral cases. All surgeries were performed by the senior author. There were 18 males and 6 females in the study group, and the mean age of the patients was 49.8 ± 14.1 years (range, 29–77). The aetiologies of the lumbosacral nonhealing wounds included the following: complications of lumbar disc herniation/lumbar spinal stenosis (LDH/LSS) surgery, 14 cases; complications of spinal tumour resection, 8 cases; and complex fracture of the spine and pelvis, 2 cases. In 16 out of 24 (66.7%) patients, internal hardware was present in the wound. Among 20 patients who had wound culture results, 16 (80%) tested positive for various types of bacteria (Additional file 1).

### Surgical characteristics and outcomes

The median flap dimension was 152 cm^2^ (range, 84–441 cm^2^). The mean surgical duration was 209.3 ± 49.9 min (range, 125–346 min). The mean follow-up time was 14.3 ± 11.4 months (range, 6–60 months). All the flaps survived successfully except in two cases in which minor complications occurred. In one case, wound dehiscence (1 cm × 1 cm) was found 2 weeks postoperatively after the stitches were removed. The wound was debrided and reclosed immediately. In the other case, a small portion of the flap was not healing well at 1 week postoperatively due to a haematoma. In this patient, the haematoma was evacuated, and a skin graft was placed under local anaesthesia. Both patients recovered uneventfully and fully healed without further sequelae (Additional file 1).

### Representative cases

#### Case 1

**Fig. 3:**
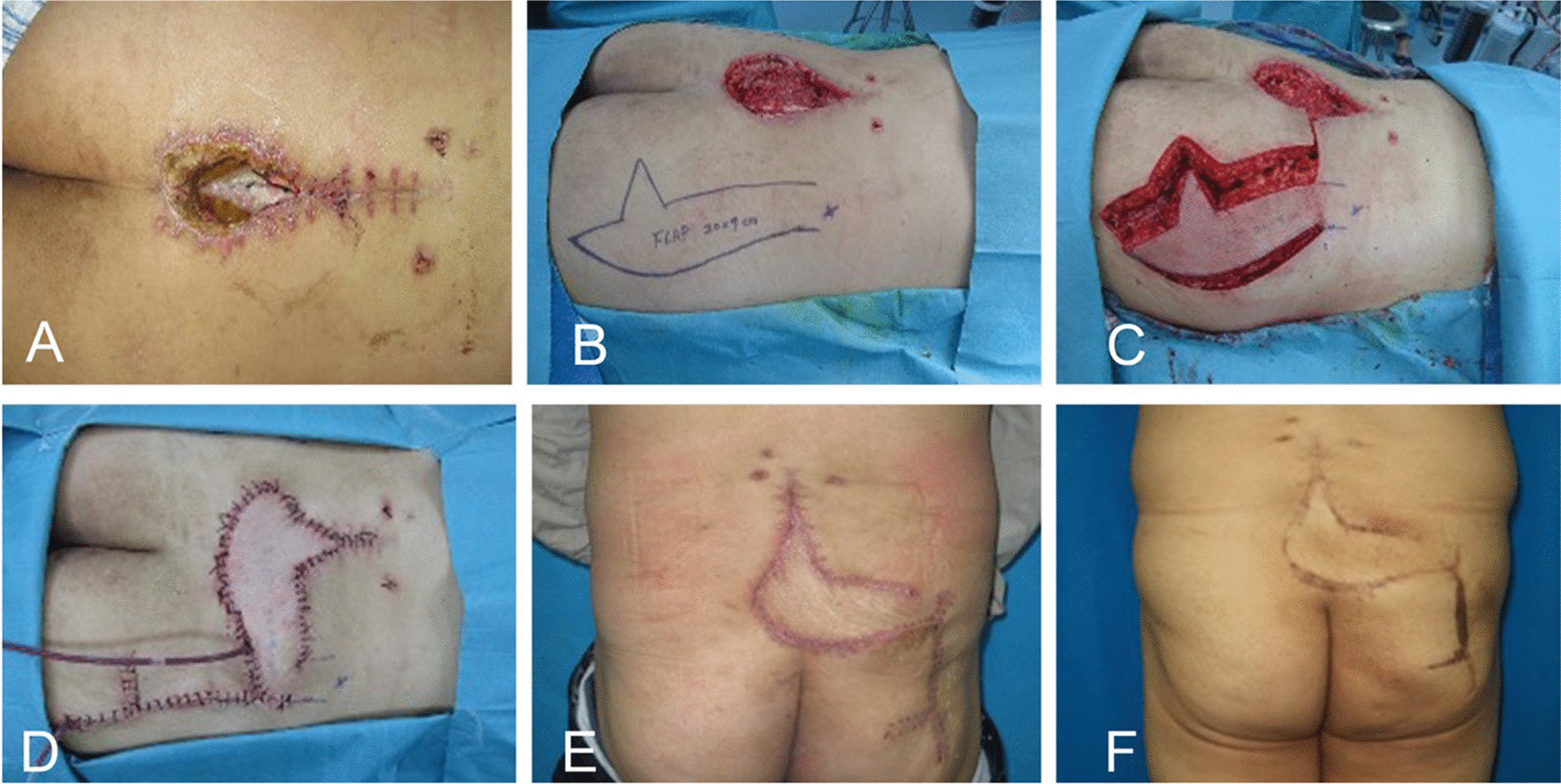
Nonhealing lumbosacral defect after lumbar spinal stenosis (LSS) surgery. **A** Posterior midline lumbosacral defect after LSS surgery (prior to debridement). **B** After wound debridement, a unilateral MFLALP flap (20 cm × 9 cm) was designed. **C**-**D** The flap was harvested and transferred to cover the wound, with primary donor-site closure. **E** Two weeks postoperatively. **F** Five years postoperatively

A 37-year-old man underwent orthopaedic surgery for lumbar spinal stenosis (L4-S1). The incision became problematic beginning at 2 weeks postoperatively. After a series of debridement and vacuum sealing drainage (VSD) procedures, the wound still showed no progress in terms of healing. MRI suggested massive liquid accumulation beneath the incision. After extensive debridement, a unilateral MFLALP flap (20 cm × 9 cm) was transferred to cover the defect (Fig. 3).

#### Case 2

**Fig. 4:**
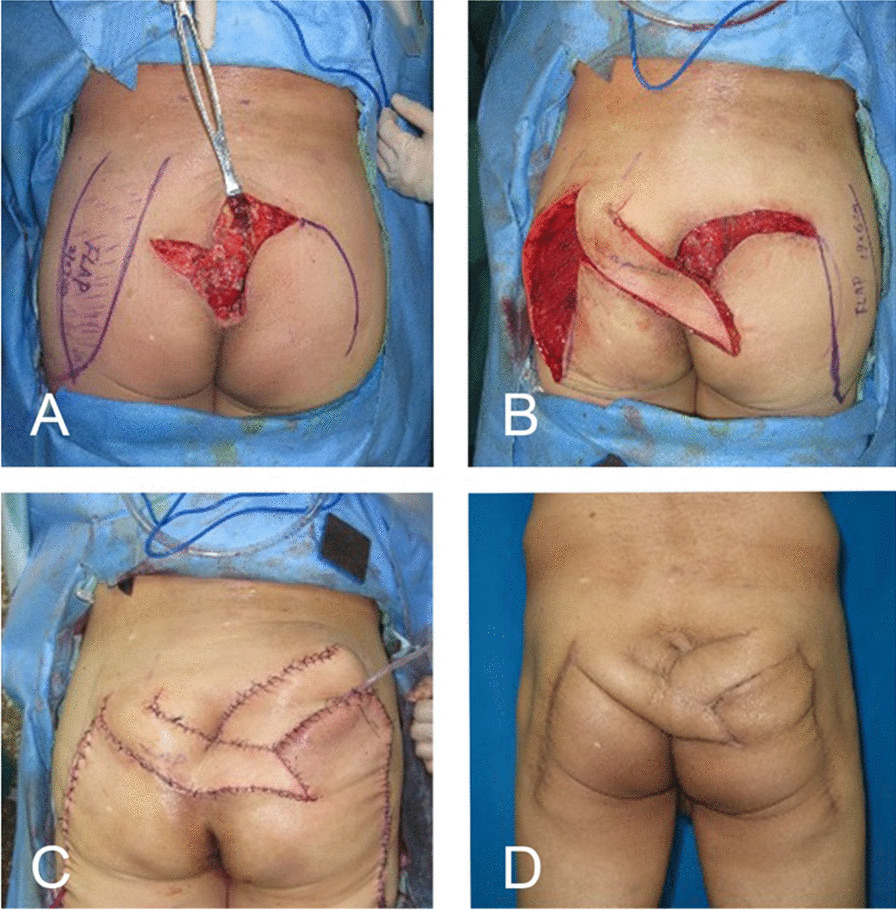
Nonhealing lumbosacral defect after chordoma removal. **A** A MFLALP flap (21 cm × 7 cm) was designed after extensive debridement. **B** The flap was transferred to the wound but was insufficient to fill the cavity. **C** A contralateral MFLALP flap (19 cm × 6 cm) was designed and harvested. Both flaps were transferred to cover the wound, with primary donor-site closure. **D** Nine months postoperatively

A 68-year-old man was diagnosed with a chordoma in the sacral area and underwent tumour removal. At 10 days postoperatively, exudation from the incision was observed. MRI showed liquid accumulation (83 mm × 105 mm × 41 mm) in the cavity created by tumour resection. After thorough debridement, reconstruction was performed using bilateral MFLALP flaps (Fig. 4).

## Discussion

### Blood supply and concept of the MFLALP flap

Kato et al. [10] studied the vascular anatomy and found four pairs of Lumbar artery perforators (LAPs) using 21 specimens of lumbar arteries in 11 cadavers. The results suggested that LAPs 1–3 all had a possibility of nonpresence, with chances of 14.3% (3/21), 9.3% (2/21) and 23.8% (5/21), respectively. In contrast, the fourth LAP was always present (21/21). Moreover, the diameter of the vascular bundle of the fourth LAP was also the largest, averaging 3.24 cm, versus 2.61 cm, 3.16 cm and 2.88 cm for the first to the third LAPs, respectively. A 3D anatomical study led by Bissell et al. [11] revealed significantly more perforators arising from the first and fourth pairs of lumbar arteries than from the second and third pairs (p < 0.05). Additionally, perforators arising from L1 and L4 have longer pedicle lengths than those arising from L2 and L3 (p < 0.05). Hamdi et al. [12] evaluated 20 female patients who underwent planned breast reconstruction involving multidetector CT, and the results showed a mean of 5 ± 2 LAPs in each patient. Although the conclusions from each study were not always consistent, likely due to the limited sample sizes and various analysis techniques and software used, they revealed that the fourth LAP was superior to the others in that it was the most reliably present and had an adequate pedicle length and diameter. In our report, using a pencil Doppler flow detector, we consistently detected the fourth LAP of all 31 flaps in 24 patients, and our favourable surgical outcomes also support the high reliability of the fourth LAP.

According to an anatomical study by Hu et al. [6], the lumbosacral area has the highest perforator density of the body, with an average of 21 perforators on each side and 5 perforators every 100 cm^2^. These perforators arise from the fourth LAP, SGAP, inferior gluteal artery perforator (IGAP) or descending branch of the sacroiliac artery, with extensive cross-circulation among these perforators [7]. Lui et al. [13], using 3D angiography based on CT scans of cadavers, suggested that the fourth lumbar artery sends one or two large branches through or in close proximity to the lumbar triangle that descend over the iliac crest to supply a segment of the superolateral gluteal region and anastomose with the ascending branch of the superior gluteal artery. An anatomic study performed by Guo et al. [9] involving 4 cadavers showed that there were massive interconnections between the LAP and SGAP at the iliac crest. Aho and his coworkers found that two major areas of lumbar perforators were clustered at 10 cm from the midline and that directly and indirectly linking vessels could be detected between the dominant lumbar artery and ipsilateral arteries in the buttock and contralateral lumbar arteries [8]. The studies above demonstrated that the extended area of the MFLALP flap was perfused through the anastomosis of the FLAP and SGAP. When the MFLALP flap was elevated, blood flowed from the FLAP to the anastomosis and then into the SGAP-dominated territory. In our report, the distal end of the MFLALP flap usually extended to the mid-lower part of the buttock, which made the territory of the donor site far beyond that of the traditional fourth LAP flap (Fig. 2).

### Advantages of the MFLALP flap

Among various reconstructive options for the coverage of lumbosacral wounds, the MFLALP flap achieves a good balance among flexibility, tissue bulk and surgical complexity. The pivot point of MFLALP flaps is based on a single perforator, which makes it more flexible for reaching wounds. Another highlight of the MFLALP flap is the tissue bulk designed at the distal part of the flap. Most lumbosacral wounds in our patient group were accompanied by dead spaces. As a result, we created a bulk design at the distal part of the flap and de-epithelized part of the distal portion to fill in the dead space [14] (Fig. 3). The trade-offs for improved volume with the design are increased potential for donor-site dehiscence and asymmetry of the buttock. There was one case of wound dehiscence in the group after the sutures were removed at 2 weeks postoperatively. A negative-pressure dressing overlying the closure may be applied for several days postoperatively to reduce the incidence of dehiscence [15]. For asymmetry, liposuction or a mini buttock lift on the contralateral side could be performed upon the patient’s request.

Traditionally, muscle flaps are given priority for wounds of concern regarding infection and hardware exposure. The paraspinous muscles may provide a reliable local reconstructive flap option for lumbar defects of many sizes; they have a reliable blood supply and avoid the need for a second donor-site incision. Unfortunately, the paraspinous musculature and/or lumbar arteries may be scarred or absent in many cases of acquired lumbar defects, making other reconstructive options necessary. The gluteus maximus muscle flap has abundant bulk for coverage. However, its use can destroy the intact gluteal muscle and sacrifice part of its function, which is crucial to ambulatory patients. The pedicle of the muscle flap is deep and short, which makes the gluteus maximus muscle flap less flexible; it is usually used as an advanced flap for the coverage of sacrococcygeal pressure sores. In addition, muscle flap dissection is usually more time consuming and involves more blood loss; therefore, it is not an ideal choice.

There have been concerns about the ability of fasciocutaneous flaps to prevent infections compared to that of musculocutaneous flaps. However, multiple studies have shown no significant differences between musculocutaneous and fasciocutaneous flaps in wound reconstruction in terms of flap survival, wound-related complications and functional recovery. Reconstructive and functional outcomes are more strongly influenced by the primary disease of the patient and condition of the wound [16, 17]. Some research has asserted that fasciocutaneous flaps may be more reliable than musculocutaneous flaps with respect to flap failure and donor-site defects [5, 18].

Fasciocutaneous flaps such as LAP and SGAP flaps are associated with less functional donor-site morbidity and can still provide a substantial volume of well-vascularized, adipofascial tissue to fill large dead spaces. The MFLALP flap is superior to the traditional LAP flap in terms of the extended flap area. Compared to SGAP flaps, MFLALP flaps also have several advantages. First, when harvesting SGAP flaps, the superior gluteal artery usually needs to be well exposed to acquire a pedicle of sufficient length. While MFLALP flaps have a longer rotation arc, the perforator pedicle can be included in the fasciocutaneous tissue instead of the perforator dissection, minimizing the operative duration and blood loss volume. Second, the pivot point of the MFLALP flap is higher than that of the SGAP flap; therefore, MFLALP flaps can cover some mid-upper lumbar wounds beyond the reach of SGAP flaps. Third, for lumbosacral wounds, the pedicle of the MFLALP flap is well outside of the wound itself and is thus less likely to be affected by prior radiation or infection. In addition, the MFLALP flap can be used as a sensory flap since the medial branch of the superior cluneal nerve runs through the osteofibrous tunnel at the posterior superior iliac crest, emerging superficially to the deep fascia 81 ± 9.2 mm from the midline and 64.7 ± 5.3 mm from the posterior superior iliac spine [19]. The superior cluneal nerve innervates the upper and middle portions of the buttock. We identified the nerve when we harvested the flap and included it in the flap in most of our cases.

For massive lumbosacral defects, when local or regional flaps cannot provide sufficient bulk, free flaps should be considered. Gaster et al. [20] reported the successful use of a free transverse rectus abdominis myocutaneous flap and SGAP vessels to reconstruct a massive irradiated sacral defect of 450 cm^2^. However, in the lumbosacral region, limitations on free flap pedicle length and a lack of receptive lumbar vessels often necessitate the use of a vein graft, which can increase both surgical complexity and flap complications.

With greater understanding of the perforasome of the posterior trunk through anatomical study as well as the development of various perforator detection devices, the reconstruction of lumbosacral wounds is entering a new era. Freestyle detection of perforators has been accomplished previously by using a Doppler probe to design the flap without preoperative knowledge of perforator locations. CT angiography and indocyanine green dye have also been utilized for the identification of possible perforator sites. DI Summa et al. [21] reported the use of freestyle perforator flaps from the paraspinal region for the reconstruction of spinal soft tissue defects. Maruccia and his coworkers [22] reported a series of 23 cases of posterior trunk reconstruction with freestyle perforator puzzle flaps. An average of 2.6 flaps were used in each patient. The authors believe that the use of multiple perforator flaps in various patterns would allow for the reconstruction of large posterior trunk defects with tension-free primary closure and minimal donor-site morbidity. Similarly, Kim et al. [23] reported the use of trilobed pedicled SGAP flaps for the coverage of lumbosacral defects to reduce the distortion and tension of the wound. Narushima et al. [24] reported the use of a lateral intercostal artery perforator-based reversed thoracodorsal artery flap for the treatment of a large radiation ulcer on the lower back. Indocyanine green dye was used to help locate the perforators and their communications. In our case series, a Doppler detector was used in all cases for perforator localization. In the future, selective LAP arteriography or indocyanine green should be used to facilitate flap design and dissection.

### Wound debridement

The management of infected wounds that also require hardware retention is challenging. The results of several investigations have suggested that positive wound culture is an independent predictor of hardware salvage failure and associated flap failure [25–27]. In our study group, 80% of patients had positive wound cultures, and 66.7% of them required hardware salvage. Therefore, effective debridement is crucial to ensure a favourable outcome. It is widely accepted that pressure irrigation is more effective than normal irrigation in terms of removing contaminants and cellular debris from wounds [28–31]. However, high-pressure irrigation is not recommended since it is associated with soft-tissue injury, can drive bacteria deeper into the wound, and does not necessarily remove more contaminants [32, 33]. When we performed surgical debridement, we utilized a 19-gauge needle adapted to a 50-ml syringe to deliver medium-pressure irrigation to the infected wounds and hardware to remove debris and bacteria. In addition to pressure irrigation, our strict debridement protocol with multiple cleansing agents and a large quantity of solution was helpful to reduce the bacterial burden. As a result, the high incidence of positive wound culture did not affect flap survival or hardware salvage.

The main limitation of our report is the small number of patients enrolled and the lack of imaging material demonstrating the anastomosis of the fourth LAP and SGAP. Furthermore, we hope to develop selective LAP arteriography and indocyanine green dye methods for preoperative and intraoperative assessment. Meanwhile, a prospective, controlled clinical trial is expected to compare the MFLALP flap with other traditional flaps to determine the effectiveness, complication rate, and postoperative quality of life yielded by our technique.

## Conclusions

In our experience, the MFLALP flap provides a reliable blood supply and favourable overall outcome. It has the advantage of a larger flap dissection area compared to that of traditional pedicled perforator flaps in this area. Therefore, the MFLALP flap is a promising alternative for the reconstruction of nonhealing lumbosacral spinal defects.

## Supplementary Information

**Additional file 1: Table S1.** Patient characteristics. **Table S2.** Operation characteristics and outcomes.

## Data Availability

All data generated or analysed during this report are included in this published article and its Additional information files.
