# Supplementary material for: Modified fourth lumbar artery local perforator flap: an alternative for reconstruction of nonhealing lumbosacral spinal defects
Source: BMC Surg. 2023 Jan 13;23:10. doi: 10.1186/s12893-023-01909-8 (PMC9840304; doi:10.1186/s12893-023-01909-8)
Supplement: Supplementary file 1 — Additional file 1: Table S1. Patient characteristics. Table S2. Operation characteristics and outcomes. [file 12893_2023_1909_MOESM1_ESM.docx]

**Table S1. Patient Characteristics**

| No | Age | Gender | Etiology | Paralysis | Internal Hardware | Comorbidity | Wound Culture |
| --- | --- | --- | --- | --- | --- | --- | --- |
| 1 | M | 29 | Trauma | Yes | Yes | - | none |
| 2 | M | 37 | LDH/LSS* surgery | No | Yes | HP | Serratia |
| 3 | F | 59 | LDH/LSS surgery | No | No | Glomerulus | - |
|  |  |  |  |  |  | nephritis |  |
| 4 | M | 39 | Trauma | Yes | Yes | ITP | E.Coli |
| 5 | M | 68 | Tumor resection | No | No | DM | Acinetobacter |
|  |  |  |  |  |  |  | aumannii |
| 6 | F | 77 | LDH/LSS surgery | Yes | Yes | HP; DM | Pseudomonas |
|  |  |  |  |  |  |  | aeruginosa |
| 7 | M | 32 | Tumor resection | No | Yes | - | Enterococcus faecalis |
| 8 | M | 37 | Tumor resection | Yes | No | - | Staphylococcus aureus |
| 9 | M | 54 | LDH/LSS surgery | No | Yes | - | - |
| 10 | F | 41 | LDH/LSS surgery | No | Yes | CAD | none |
| 11 | M | 37 | LDH/LSS surgery | No | Yes | - | Staphylococcus aureus |
| 12 | M | 72 | Tumor resection | No | No | HP | none |
| 13 | M | 52 | LDH/LSS surgery | No | Yes | DM | Staphylococcus aureus |
| 14 | F | 39 | LDH/LSS surgery | No | Yes | - | E.Coli |
| 15 | M | 58 | Tumor resection | No | No | - | - |
| 16 | M | 36 | LDH/LSS surgery | No | Yes | - | Coagulase-negative |
|  |  |  |  |  |  |  | staphylococcus |
| 17 | F | 48 | Tumor resection | Yes | No | - | - |
| 18 | M | 44 | LDH/LSS surgery | No | Yes | - | Staphylococcus aureus |
| 19 | M | 67 | LDH/LSS surgery | No | Yes | DM | Enterobacter cloacae |
| 20 | M | 33 | LDH/LSS surgery | No | Yes | - | Pseudomonas |
|  |  |  |  |  |  |  | aeruginosa |
| 21 | M | 52 | Tumor resection | No | No | - | Staphylococcus |
|  |  |  |  |  |  |  | Epidermidis |
| 22 | F | 70 | LDH/LSS surgery | No | Yes | Rheumatoid | none |
|  |  |  |  |  |  | arthritis |  |
| 23 | M | 66 | LDH/LSS surgery | No | Yes | HP; DM | Enterobacter cloacae |
| 24 | M | 47 | Tumor resection | Yes | No | - | Staphylococcus aureus |

**Table S2. Operation Characteristics and Outcomes**

| No | Unilateral/  Bilateral | Defect size  (cm^2^) | Flap size  (cm^2^) | Operative time (min) | Complication | | Management | | Follow up time (mos) | |  |
| --- | --- | --- | --- | --- | --- | --- | --- | --- | --- | --- | --- |
| 1 | UL | 98 | 152 | 238 | | - | | - | | 8 | |
| 2 | UL | 160 | 220 | 210 | | - | | - | | 60 | |
| 3 | UL | 144 | 180 | 210 | | Wound dehiscence | | Debridement, re-closure | | 16 | |
| 4 | UL | 156 | 238 | 346 | | Hematoma | | Hematoma evacuation, skin graft | | 6 | |
| 5 | BL | 102; 150 | 144; 188 | 223 | | - | | - | | 9 | |
| 6 | BL | 84; 96 | 136; 144 | 245 | | - | | - | | 6 | |
| 7 | UL | 144 | 180 | 155 | | - | | - | | 6 | |
| 8 | UL | 96 | 136 | 130 | | - | | - | | 12 | |
| 9 | UL | 144 | 181 | 170 | | - | | - | | 14 | |
| 10 | BL | 60; 72 | 84; 98 | 225 | | - | | - | | 8 | |
| 11 | UL | 126 | 176 | 196 | | - | | - | | 21 | |
| 12 | BL | 96; 98 | 140; 144 | 267 | | - | | - | | 12 | |
| 13 | UL | 112 | 152 | 230 | | - | | - | | 7 | |
| 14 | UL | 121 | 144 | 205 | | - | | - | | 9 | |
| 15 | UL | 168 | 210 | 135 | | - | | - | | 6 | |
| 16 | UL | 256 | 313 | 240 | | - | | - | | 18 | |
| 17 | BL | 84; 96 | 102; 133 | 264 | | - | | - | | 14 | |
| 18 | UL | 96 | 144 | 195 | | - | | - | | 11 | |
| 19 | UL | 116 | 152 | 146 | | - | | - | | 30 | |
| 20 | UL | 176 | 207 | 125 | | - | | - | | 6 | |
| 21 | UL | 361 | 441 | 181 | | - | | - | | 25 | |
| 22 | BL | 96; 84 | 160; 126 | 255 | | - | | - | | 17 | |
| 23 | UL | 156 | 209 | 208 | | - | | - | | 6 | |
| 24 | BL | 91; 112 | 133; 144 | 223 | | - | | - | | 15 | |
